# Serum GDF15, a Promising Biomarker in Obese Patients Undergoing Heart Surgery

**DOI:** 10.3389/fcvm.2020.00103

**Published:** 2020-06-24

**Authors:** Shreya Sarkar, Stephanie Legere, Ian Haidl, Jean Marshall, Jeffrey B. MacLeod, Christie Aguiar, Sohrab Lutchmedial, Ansar Hassan, Keith R. Brunt, Petra Kienesberger, Thomas Pulinilkunnil, Jean-François Légaré

**Affiliations:** ^1^New Brunswick Heart Centre, Saint John, NB, Canada; ^2^Dalhousie Medicine New Brunswick, Saint John, NB, Canada; ^3^IMPART Investigator Team Canada, Saint John, NB, Canada; ^4^Department of Microbiology and Immunology, Dalhousie University, Halifax, NS, Canada; ^5^Department of Pharmacology, Dalhousie University, Halifax, NS, Canada; ^6^Department of Biochemistry, Dalhousie University, Halifax, NS, Canada

**Keywords:** BMI, outcomes, cardiac surgery, heart failure, metabolism, obesity

## Abstract

**Background:** Obesity is a risk factor that negatively impacts outcomes in patients undergoing heart surgery by mechanisms that are not well-defined nor predicated on BMI alone. This knowledge gap has fuelled a search for biomarkers associated with cardiovascular diseases that could provide clinical insight to surgeons. One such biomarker is growth differentiation factor15(GDF15), associated with inflammation, metabolism, and heart failure outcomes but not yet examined in the context of obesity and cardiac surgery outcomes.

**Methods:** Patients undergoing open-heart surgery were consented and enrolled for blood and tissue (atria) sampling at the time of surgery. Biomarker analysis was carried out using ELISA and western blot/qPCR, respectively. Biomarker screening was classified by inflammation(NLR, GDF15, Galectin3, ST2, TNFR2), heart failure(HF)/remodeling(NT-proBNP) and metabolism(glycemia, lipid profile). Patients were categorized based on BMI: obese group (BMI ≥30.0) and non-obese group(BMI 20.0–29.9). Subsequent stratification of GDF15 high patients was conservatively set as being in the 75th percentile.

**Results:** A total of 80 patients undergoing any open-heart surgical interventions were included in the study. Obese (mean BMI = 35.8, *n* = 38) and non-obese (mean BMI = 25.7, *n* = 42) groups had no significant differences in age, sex, or co-morbidities. Compared to other biomarkers, plasma GDF15 (mean 1,736 vs. 1,207 ng/l, *p* < 0.001) was significantly higher in obese patients compared to non-obese. Plasma GDF15 also displayed a significant linear correlation with BMI (*R*^2^ = 0.097; *p* = 0.0049). Atria tissue was shown to be a significant source of GDF15 protein and tissue levels significantly correlated with plasma GDF15 (*R*^2^ = 0.4, *p* = 0.0004). Obesity was not associated with early/late mortality at median follow-up >2years. However, patients with high GDF15 (>1,580 ng/l) had reduced survival (65%) compared to the remaining patients with lower GDF15 levels (95%) by Kaplan Meier Analysis (median >2 years; *p* = 0.007).

**Conclusions:** Circulating GDF15 is a salient biomarker likely sourced from heart tissue that appears to predict higher risk obese patients for adverse outcomes. More importantly, elevated GDF15 accounted for more sensitive outcome association than BMI at 2 years post-cardiac surgery, suggesting it heralds links to pathogenicity and should be actively studied prospectively and dynamically in a post-operative follow-up.

**Trial number:** NCT03248921.

## Introduction

Obesity rates have doubled since 1980 and affect >20% of the North American population (1). Our health-care system is negatively impacted by the growing association between obesity and chronic health conditions like hypertension, diabetes, and cardiovascular disease (2). In particular, invasive cardiovascular interventions like heart surgery that are resource-intensive are increasingly indicated in obese patients that face higher risks of complication or negative outcomes (3).

Geographical demographics also play into clinical management and resource allocations, with recent estimates from Atlantic Canada showing that >35% of patients undergoing heart surgery are obese (4). Body mass index (BMI), the most commonly used measure to define overweight (BMI 25.0–29.9 kg/m^2^) and obesity (>30.0 kg/m^2^) is used in an effort to guide a standardized approach to outcomes but has not been effective at segregating negative outcomes within the obese population. Indeed, the relationship of BMI can be U-shaped with extremes of BMI having worse outcomes (5). Studies have acknowledged this complex relationship and suggest that traditional measures of obesity such as BMI incompletely predict outcomes given the heterogeneity in BMI associated pathology. Determining other details reliably with respect to lean body/adipose tissue distribution and assessing functional impairments are also limited and time consuming (6, 7). Prior studies yield conflicting results as to how obesity impacts postoperative outcomes (6, 7).

Taken together, biomarkers could be used clinically to better characterize the role of obesity in influencing cardiovascular outcomes. GDF15 has been suggested as a potential candidate and is associated with obesity, body weight regulation, and heart failure (8). GDF15 is a member of the TGFβ superfamily, upregulated under stress or following injury (9); appearing to be important in regulating inflammation (9). In patients undergoing heart surgery, elevated GDF15 can predict worse outcomes related to myocardial or renal injury (8) but has also been negatively associated with risk of atrial fibrillation (10). It is not known whether GDF15 is associated with obesity-related outcomes. In the present study, we sought to characterize the clinical characteristics and outcomes of patients undergoing elective heart surgery in which blood and tissue samples were assessed for biomarkers, including GDF15 in relation to obesity outcomes. Additional biomarkers tested in pre-surgery plasma were related to inflammation (NLR, GDF15, Galectin3, ST2, TNFR2), heart failure (HF)/remodeling (NT-proBNP) and metabolism (glycemia, lipid profile).

## Methods

### Study Population and Objectives

Consecutive patients were enrolled prospectively based on the requirement for elective open-heart surgery at the New Brunswick Heart Centre (NBHC) in Saint John, New Brunswick. Approval from the Research Ethics Board (REB) was obtained from Horizon Health Network prior to the study (11).

All consented participants received the standard of care in terms of treatment. The exclusion criteria included the need for urgent or emergent surgery, patients with BMI <18.5 kg/m^2^ and patients older than 75 years of age to limit the effects of advanced age and/or frailty. The aims were to characterize obesity in patients undergoing cardiac surgery and allow select measurements of obesity, testing of exercise capacity and functional status, and blood and tissue sampling to allow profiling of circulating biomarkers and immune-metabolic status.

Patients were divided into groups based on BMI as obese (BMI ≥30.0 kg/m^2^) and compared to non-obese (BMI 18.5–29.9 kg/m^2^). A detailed classification included: patients of normal weight (BMI of 18.5–24.9 kg/m^2^), which served as the controls, pre-obese (BMI 25.0–29.9), obese class I (BMI 30.0–34.9), class II (BMI 35.0–39.9), and class III (BMI ≥40.0).

### Study Procedures

Preoperative blood (fasted pre-op) was collected (4 ml) from all patients, 30 min before surgery, following induction of anesthesia in separate tubes (VWR, USA) for plasma and serum, centrifuged at 4°C for 15 min at 2,000 × g to separate plasma/serum and stored at −80°C until further analysis. A portion of right atrial appendage was excised from the patients during surgery. The atrium was divided into two portions. 1/3rd of the tissue was fixed in 10% formalin for histology, while the remaining 2/3rd was snap-frozen for analysis of RNA and proteins. Further details are given in the following sections.

In all patients, cardiac surgery was performed with cardiopulmonary bypass and anticoagulation was achieved using intravenous heparin (400 IU/kg) with a target activated clotting time (ACT) >450 s. Antifibrinolytic agents were given to all patients and consisted mainly of tranexamic acid. Intermittent cold blood cardioplegia was delivered in an antegrade or retrograde fashion based on surgeons' preference. Protamine sulfate was given for the reversal of heparin in all patients. Patients also received routine baseline 12-lead electrocardiograms upon admission to the surgical intensive care unit (SICU). Resumption of routine postoperative medications occurred as indicated and included anti-platelet agents within 24 h, statins and β-blockers. All patients were monitored during their stay in- hospital.

### Variable Selection

Preoperative clinical characteristics of interest included age, gender, New York Heart Association (NYHA) functional class, urgency of surgery (urgent if required within 24 h, in-hospital urgent if the patient required hospitalization until the time of surgery, and elective or outpatient) and diabetes. Intraoperative variables included pump time and clamp time. Standard blood analysis included complete blood counts, neutrophil-to-lymphocyte ratio (NLR) and peak troponin (collected within 24 h of surgery).

Clinical outcomes of interest were divided into in-hospital outcomes and long-term outcomes after discharge. In-hospital outcomes included mortality, length of hospitalization (in days), discharge disposition (home or other institution), cerebrovascular insult (transient or permanent), and new-onset atrial fibrillation (any duration during the hospitalization). The long-term outcome also included mortality, median time to last follow-up (days), NYHA class at the time of last follow-up and the use of loop diuretics at the time of the last follow-up.

All patient information, procedural details, and outcomes were collected and maintained in our New Brunswick Heart Center Cardiac Surgery Registry (4). Routine follow-up was standardized and included discharge echocardiography for valve patients or otherwise indicated and a routine follow-up 6 weeks after surgery. Long-term outcomes beyond 6 weeks were obtained from a combination of telephone interviews (standardized approach to questions), medical records and diagnostic imaging datasets.

### Biomarker Analysis Using Luminex Assay

Pre-operative plasma (1:10) was used for the detection of biomarkers according to manufacturer's protocol using Luminex assay with ProcartaPlex Human Basic Kit (Cat. # EPX010-10420-901, Thermo Fisher Scientific, MA, USA) and Human GDF-15 Luminex Performance Assay (Cat. # LUCAM975, R&D Biosystems, MN, USA). Fluorescence-coded magnetic microparticles coated with antibodies specific for the desired cytokines and chemokines (Table 1) were used to assay plasma and read using a Bio-Rad Bio-Plex dual laser (BioRad, Mississauga, Canada). Luminex assays were carried out using a batched sample approach and included repeat samples to ensure that findings from assays done on two separate dates were comparable. Levels of GDF15 and TG were separated based on the 75th percentile for further analysis (12).

**Table 1:** Biomarkers used for analysis.

CCL2	Galectin-3	GDF-15	IL-18	MMP-2
MMP-9	NT-proBNP	sST2	TNFR2	VEGF-A

### Protein Expression Using Western Blot

Right atrial appendage (~30 mg, *n* = 5 per obesity group) were analyzed by western blot. Proteins were isolated by homogenizing tissues in lysis buffer as detailed previously (13). In brief, frozen tissues were powdered and homogenized in ice-cold lysis buffer containing a protease and phosphatase inhibitor cocktail and centrifuged at 15,000 × g to obtain whole cellular proteins. Protein concentration was determined by BCA protein assay (Pierce, Thermo Fisher Scientific, MA, USA), electrophoresed in a 10% sodium dodecyl sulfate-polyacrylamide (SDS) gel, transferred to a nitrocellulose membrane and visualized by a reversible stain (MemCode Reversible protein Stain, Pierce, Thermo Fisher Scientific, MA, USA). GDF15 was probed using the primary antibody anti-Mic-1 (3294, Cell Signaling Technology) and the blots were developed using Western Lightning Plus-ECL enhanced chemiluminescence substrate (Perkin Elmer, MA, USA). Image Lab software was used for densitometric analysis (Bio-Rad) and GDF15 protein levels were normalized to total proteins.

### RNA Expression

RNA expression of GDF15 was analyzed in a total of 25 samples based on RNA quality; normal (*n* = 5), pre-obese (*n* = 5), obese class I (*n* = 5), class II (*n* = 5), and class III (*n* = 5). RNA was isolated from atrial tissue (~30 mg) using Ribozol (Amresco, OH, USA), followed by chloroform extraction (13). RNA integrity was assessed in all cases (Synergy H4, Biotek, VT, USA), and 1 μg of RNA was used to synthesize cDNA (qScript cDNA supermix, Quanta Biosciences). For quantitative Polymerase Chain Reaction (qPCR), 2 μl of cDNA was mixed with SYBR Green PCR supermix (AB1323A, Thermo Fisher Scientific) and primers (Human GDF15 Forward 5′-CTCCAGATTCCGAGAGTTGC-3′ and Reverse 5′-AGAGATACGCAGGTGCAGGT-3′) and the reaction was performed in duplicate with 40 cycles (13). Human PPIA (Forward 5′-ATGTGTCAGGGTGGTGACTTC-3′ and Reverse 5′-GCCATCCAACCACTCAGTCTT-3′) was used as a reference gene and results expressed as a ratio of Cycle threshold (Ct) values of the target and reference genes.

### Histology and Immunohistochemistry

Representative atrial appendage samples (*n* = 2 for each group of normal, pre-obese, obese class I, class II, and class III patients) were used for GDF15 protein expression and localization. Paraffin sections (5 μm) were deparaffinized, serially hydrated and stained with Hematoxylin and Eosin for histology (14). Sections from the same tissue were then used for immunostaining of GDF15. In brief, paraffin sections were hydrated, antigens retrieved using Citrate buffer, blocked with 15% goat serum and incubated with anti- GDF15 primary antibody at 1:100 dilution (Santacruz Biotechnology) (14). After incubation with Horse Radish Peroxidase (HRP)- conjugated secondary antibody (1:500), slides were developed with 3,3′ Diaminobenzidine (DAB) (Sigma Millipore, MA, US) and counter-stained with Hematoxylin. Negative control was incubated without the primary antibody. Images were photographed using a bright field microscope (Optika, Italy).

### Data Analysis

Data analysis was performed using GraphPad Prism 6 (GraphPad Software Inc, La Jolla, CA). Categorical variables were reported as percentages and analyzed by Chi-square. Continuous variables were analyzed by Student *t*-test. Pearson's correlation followed by linear regression was used to correlate variables. Survival was determined by Kaplan Meier curves, followed by Mantel-Cox Log-rank test. A *p* < 0.05 was considered statistically significant.

### Ethics

This study was conducted with the full approval of the institutional (Horizon Health Network) Research Ethics Board. All personal identifiers were stripped before data analysis to ensure patient anonymity and confidentiality.

## Results

### Study Population

A total of 80 consecutive patients undergoing elective cardiac surgery were included in the study (Table 2). Patients were initially categorized as non-obese (*n* = 38) and obese (*n* = 42) with similar characteristics and no significant differences in age, sex, co-morbidities, and type of procedure performed. However, obese patients were more likely to have more advanced NYHA (class III-IV) symptoms and more limited exercise capacity (6 min walk test) before surgery. Patients were mostly male (80%) with a mean age of 63.5 years and good heart function (mean EF = 59.8%). All patients underwent elective heart surgery with 55% of cases being isolated CABG surgery, 19% isolated valve surgery, and 26% undergoing combined valve and CABG surgery.

**Table 2:** Patient characteristics.

**Characteristics**		**Non-obese (*n* = 38)**	**Obese (*n* = 42)**	***p*-value**
Age		64.0 + 1.3	63.0 + 1.0	0.52
Female sex		20%	21%	0.79
BMI		25.7 + 0.5	35.8 + 0.7	**<0.0001**
	*Normal*	15	0	
	*Pre-obese*	23	0	
	*Class I*	0	17	
	*Class II*	0	16	
	*Class III*	0	9	
Diabetes		26.3%	45.2%	0.1
HTN		73.7%	78.6%	0.79
Dyslipidemia		60.5%	78.6%	0.09
PVD		2.6%	11.9%	0.2
CVD		12.5%	9.5%	1
Renal failure		0%	2.30%	1
EF		61.8 + 1.6	58.0 + 1.9	0.12
Atrial fibrillation		13.2%	11.9%	1
NYHA (Median)		2 (1–3)	3 (1–3)	0.5
6 min walk (meters)		351.5 + 21.1	285.6 + 15.2	**0.014**
Procedure	Isolated CABG	63.2%	47.6%	0.46
	Isolated Valve	18.4%	19.0%	
	Other	0.0%	2.4%	
	Combined	21.1%	31.0%	

*BMI, body mass index; HTN, hypertension; PVD, Peripheral Vascular Disease; CVD, Cardiovascular disease; EF, ejection fraction; NYHA, New York Health Association; CABG, coronary artery bypass graft*.

*Bold values indicate significant p-values*.

### Significant Correlation Between Increasing Obesity and Circulating GDF15 Levels

**Figure 1:**
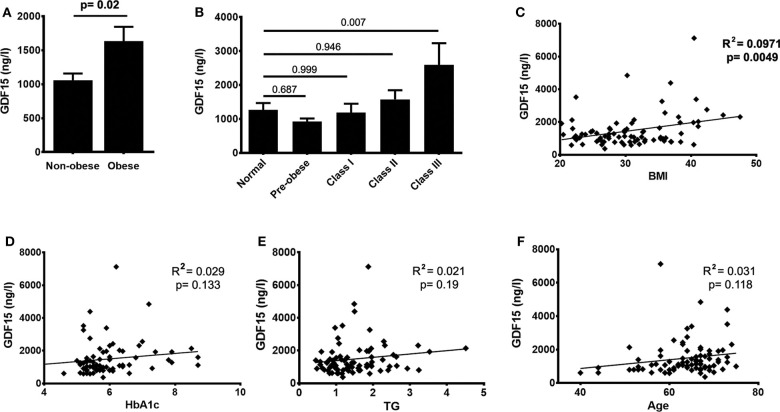
GDF15 significantly correlates with obesity but not with metabolic status of patients. **(A)** Levels of GDF15 in the plasma are increased in obese patients compared to non-obese patients. **(B)** GDF15 increased in obesity classes II and III, with a significant difference between normal and class III patients. **(C)** Circulating GDF15 also significantly correlates with body mass index (BMI), but not with glycated hemoglobin (HbA1c), triglycerides or age **(D–F)**. Data were analyzed using unpaired *t*-test, one-way ANOVA, Pearson's correlation and regression.

Biomarkers analyzed in pre-surgery plasma were categorized based on function: inflammation-related (NLR, GDF15, Galectin3, ST2, TNFR2), heart failure (HF)/remodeling related (NT-proBNP) and metabolism-related (glycemia, lipid profile) (Table 3). In particular, GDF15 levels were significantly higher in obese patients (Figure 1A). This suggests that inflammation may be important in obese patients, but this was not replicated with other inflammatory biomarkers. Triglyceride levels were significantly higher in obese patients, suggesting metabolic maladaptation independent of glycemia, since random blood sugar (RBG) and HbA1c were not different between obesity groups. Plasma GDF15 levels, when normalized to BMI categories displayed a severity-dependent relationship with obesity having the highest GDF15 in obesity Class-III patients (Figure 1B), something we did not see with triglycerides (data not shown). GDF15 also significantly correlated with BMI (*R*^2^ = 0.097, Figure 1C). However, GDF15 expression did not appear to correlate with nutrient-related abnormalities, such as hyperglycemia (defined by HbA1c and RBG, Figure 1D), hyperlipidemia (defined by TG and TC, Figure 1E) or age (Figure 1F). GDF15 more than any other factors were higher amongst obese patients, providing an opportunity to be a salient indicator associated with obesity-related outcomes.

**Table 3:** Biomarker findings.

**Characteristics**		**Non-obese (*n* = 38)**	**Obese (*n* = 42)**	***p*-value**
Inflammation	NLR	3.4 + 0.3	4.3 + 0.8	0.36
	GDF15	1,207 + 89.30	1,736 + 202.2	**<0.001**
	Galectin 3	27,599 + 2,549	30,891 + 2,314	0.34
	ST2	23,435 + 2,272	29,557 + 3,733	0.18
	TNFR2	2,604 + 261.9	2,953 + 211.1	0.3
CHF/remodeling	NT-proBNP	1,215 + 121.8	1,130 + 91.7	0.57
	Atrial fibrosis	29.7 + 4.4	35.3 + 3.8	0.33
	LVEDP	15.2 + 1.2	20.1 + 1.9	**0.032**
Metabolic	Random BG	6.9 + 0.6	7.8 + 0.4	0.22
	HbA1c	5.7 + 0.1	6.0 + 0.2	0.27
	TG	1.2 + 0.1	1.8 + 0.1	**<0.0001**
	TC	717.0 + 72.2	766.8 + 111.0	0.71

*CHF, chronic heart failure; BG, blood glucose; TG, triglyceride; TC, total cholesterol; LVEDP, Left Ventricular End-Diastolic Pressure*.

*Bold values indicate significant p-values*.

### Atrial Tissue Expression of GDF15

**Figure 2:**
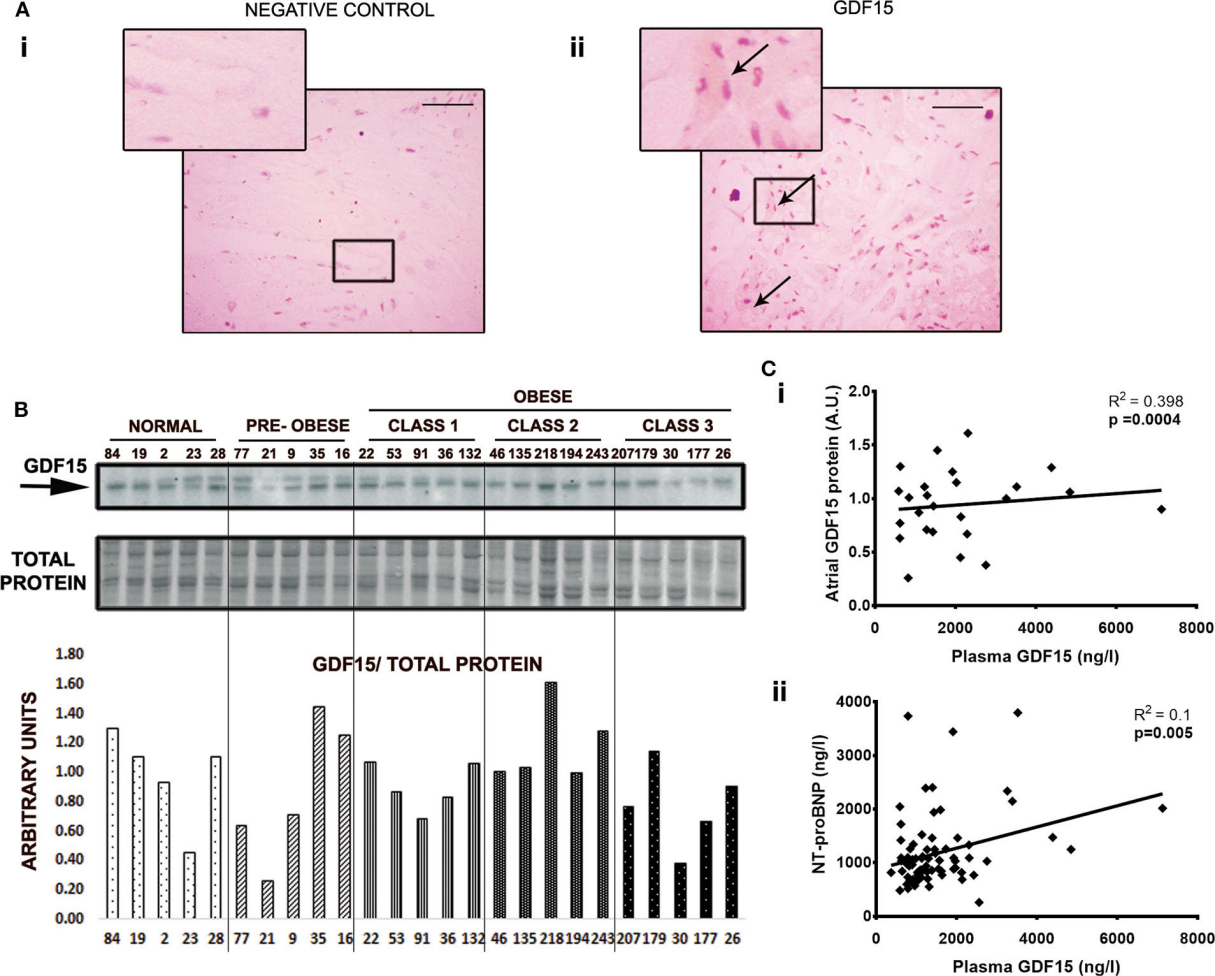
GDF15 protein expression in atrial tissues (AA) correspond with circulating GDF15. **(A)** Low to moderate cytoplasmic expression of GDF15 was observed in atrial tissues. **(i)**, Negative control. **(ii)**, GDF15 immunohistochemistry. **(B)** GDF15 showed weak protein expression in atrial tissue. GDF15 protein was normalized to whole protein stain. **(C)** Plasma GDF15 correlates with protein expression **(i)**, and with NT-proBNP, a marker for heart failure **(ii)**. Data were analyzed using Pearson's correlation and linear regression. For immunochemistry, outer magnification: 40X, scale bar: 50 μm. Arrows represent protein stain.

Representative tissue samples from each obesity group were analyzed for GDF15 by western blot and immunohistochemistry (Figures 2Ai,ii). GDF15 protein was detected in all atrial samples but was not significantly different between obesity groups (Figure 2B). Atrial tissue expression of GDF15 correlated significantly with plasma GDF15 (*p* < 0.001, *R*^2^ = 0.398) (Figure 2Ci). Together these findings suggest that atrial tissue may be a significant source of circulating GDF15. One should note that atrial GDF15 mRNA expression was unreliable in some samples or otherwise very low (Supplementary Figure 1A) and did not correlate with plasma GDF15 protein (Supplementary Figure 1Bi) or atrial GDF15 protein (Supplementary Figure 1Bii), highlighting the importance of assessing both mRNA transcript and protein. Our findings were supported by immunohistochemical studies, which showed GDF15 cytoplasmic expression in atrial tissues (Figure 2A). GDF15 levels in cardiac adipose tissue were not detectable in most patients (unpublished results) supporting our findings that atrial (cardiac) tissue expression could be an important source of plasma GDF15.

### Obesity Severity Did Not Affect Clinical Outcomes or Long-Term Survival

All patients were followed-up after surgery for a minimum of 30 days post-discharge (Table 4). The overall in-hospital mortality rate was 3.8% and the median length of hospitalization of 5 days (IQR 5-7). The overall outcomes did not appear to differ significantly between non-obese and obese patients, except for obese patients more likely to require longer hospitalization (median 5 vs. 6 days; *p* = 0.003). Similarly, long-term survival was similar between non-obese and obese patients at a median follow-up of 24 months (IQR 20-28) with total mortality of 8.8%.

**Table 4:** Patient outcomes.

**Characteristics**		**Non-obese (*n* = 38)**	**Obese (*n* = 42)**	***p*-value**
**In-hospital outcome**				
*Mortality*		0% (*n* = 0)	7.1% (*n* = 3)	0.24
*Median LOS*		5 (4–6)	6 (6–7)	**0.003**
*Discharge disposition*	Home	94.7%	79.5%	0.087
	Other institution	5.3%	20.5%	
*CVA*		0% (*n* = 0)	4.8% (*n* = 2)	0.49
*Afib*		31.6 (*n* = 12)	40.5 (*n* = 17)	0.49
**Long-term outcome**				
*Mortality*		5.3% (*n* = 2)	11.9% (*n* = 5)	0.44
*Median follow-up*		27 (24–29)	21 (19–24)	**0.008**
*NYHA (III-IV)*		8.00%	13.80%	0.67
*Loop diuretics*		0.0%	7.1%	0.24

*LOS, Length of stay; CVA, cardiovascular accident; Afib, atrial fibrillation; NYHA, New York Heart Association*.

*Bold values indicate significant p-values*.

### Elevated Plasma GDF15 Significantly Correlated With NT-ProBNP Levels Before Surgery and Corresponded With a Poor Long-Term Prognosis

**Figure 3:**
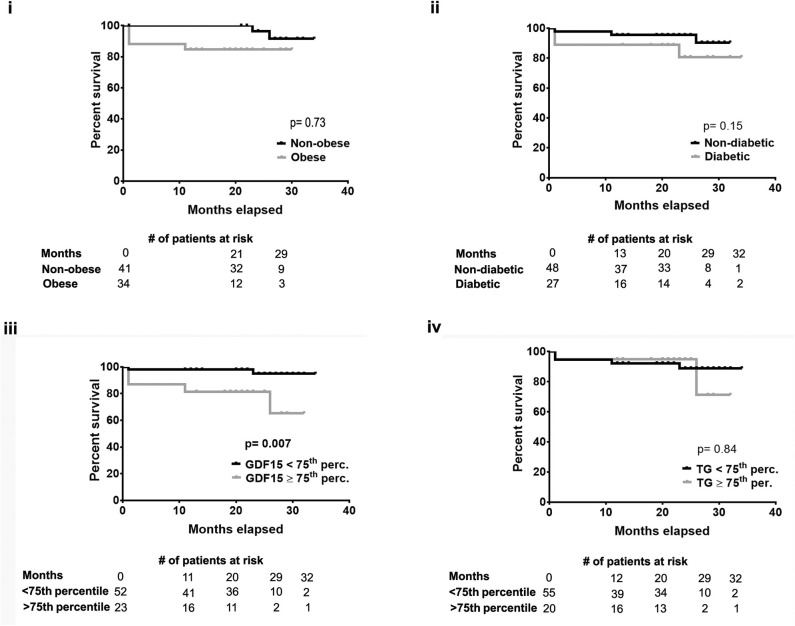
Patients with GDF15 ≥75th percentile had a significantly worse prognosis. Using Kaplan Meier curves, followed by Log-Rank test, there was no significant difference in 2 year post-surgery survival between patients who were **(i)** non-obese/obese **(ii)** non-diabetic/diabetic **(iv)** or TG <75th percentile/≥75th percentile. However, patients with plasma GDF15 ≥75th percentile had a significantly poor post-surgery outcome compared to those with GDF15 <75th percentile **(iii)**.

Heart function assessed by ejection fraction (EF) was 59.8% (±11.1%) for the entire cohort. However, some patients suffered from CHF symptoms as defined by NYHA III-IV before surgery and were considered to have HF with preserved EF (HFpEF). Using this definition, HFpEF was present in 46% of patients and slightly more common in obese patients (55 vs. 37%; *p* = 0.108) without reaching significance. With additional analysis using NT-proBNP as a biomarker of HF, we were not able to detect a significant difference between obesity groups. However, GDF15 correlated significantly with NT-proBNP (*R*^2^ = 0.09, *p* = 0.005, Figure 2Cii) linking GDF15 to remodeling and CHF. Similar long-term survival in obese and non-obese patients post-cardiac surgery suggests that obesity defined by BMI does not affect survival in our population (Figure 3i). Similarly, there was no significant difference in survival between diabetic/non-diabetic patients (Figure 3ii). Given the novel association between obesity severity and GDF15, we sought to evaluate the clinical implications of an elevated GDF15 to better define at risk patients. To achieve this, we grouped patients based on median/IQR using the 75th percentile as the cut off (GDF15 = 1,580). Using this approach, 24 patients were found to have GDF15 ≥75th percentile (mean GDF15 2626.8 ng/L) vs. 56 patients were GDF15 <75th percentile group (mean GDF15 995.43 ng/L, *p* < 0.001). Using this approach, we were able to demonstrate that GDF15 ≥75th percentile group have a significantly worse survival at 2 years after surgery compared to patients in the GDF15 <75th percentile group (*p* = 0.007) (Figure 3iii; the clinical characteristics of the patients grouped this way is in Supplementary Table 1). Survival was not different if patients were grouped similarly based on TG (Figure 3iv). This indicates that GDF15, while obesity-associated in part, can also identify significant differences with regard to outcomes over classical case histories associated with metabolic disease.

## Discussion

At a population level, obesity is associated with a higher risk of developing cardiovascular morbidity and mortality (15). This impacts the patient treatment strategies for obese patients requiring cardiac surgery (16). Work by our group has shown that obesity is independently associated with increased morbidity after cardiac surgery and this results in increased resource utilization (4). Recent studies suggest the association of several biomarkers with BMI and chronic heart failure (17). However, to date there has been little information on specific biomarkers that could be used to better stratify outcome in the obese population. The goal of our work was to evaluate biomarkers that could do this.

Here we examined the novel circulating biomarker GDF15 in cardiac surgery patients with heart disease given the suggested pre-clinical role of GDF15 in obesity (18). Our study is supported by others, suggesting that higher GDF15 expression predicted worse outcomes in heart surgery patients (19), but its relationship to obesity merited further investigation. In the present study, we show that serum GDF15 expression is higher in the most obese of patients undergoing cardiac surgery. This was further supported by a linear correlation between BMI and GDF15 levels. Taken together, our findings suggest for the first time that GDF15 could not only be an important biomarker in cardiovascular disease but may also help determine the impact of obesity on cardiovascular outcomes.

The clinical characteristics of non-obese and obese patients appeared similar to allow comparison. However, these were small groups of non-randomized patients and given this sample size, significant differences may be under-appreciated. It is also important to note that a multivariable analysis was not possible given the small size of the study. As such, this study should be considered carefully and observational as a means of supporting further investigations. Specifically, in our study population there were no significant differences regarding age, gender, diabetes, peripheral vascular disease (PVD) or EF, all of which could potentially impact GDF15 expression and are themselves known risk factors negatively impacting outcomes. We only noted that obese patients appeared functionally more limited based on higher NYHA (II vs. III) and 6 min walk test (285.6 vs. 351.5 m; *p* = 0.012). Previous work suggests that physical activity can increase GDF15 expression in an acute setting (20). This is contrary to our findings in which obese patients had lower ability for activity but higher GDF15 levels and likely reflects the methodology or biology associated with sampling GDF15 following exercise. Likely, the physical activity of patients waiting for cardiac surgery is sedentary and as such may not have skewed toward the efforts of daily activity to influence our results. Future studies should explore GDF15 prospectively through follow-up, particularly with sampling through cardiac rehabilitation.

We highlighted in the present manuscript the importance of GDF15, but a number of additional biomarkers were also examined without the same degree of salience given the small sample size. GDF15 and TG levels were the only two biomarkers that were significantly higher in obese patients. We were not able to demonstrate a similar incremental response relationship between TG and obesity. The lack of association between GDF15 and TG or blood sugar suggests they are unrelated to circulating GDF15 levels in obesity. This could also indicate that structural alterations in the patient more than metabolic alternations are driving higher GDF15 and could be further studied in experimental models of disease. Our findings are in contrast to some reports correlating GDF15 to TG, HbA1c in type 2 diabetes and elderly patients (21, 22). We interpret our findings to highlight the complexity of viewing obesity primarily from a BMI point of view given the high variability between individuals, where obesity can still express significant nutritional/metabolic abnormalities that we could not demonstrate in our study. However, although metabolic syndrome could explain some of our findings, our small sample size makes it difficult to rule out the possibility by multivariate corrections to blood pressures, age, and other risk factors at this time. A focused study on GDF15 could reveal additional information, either clinically for through experimental models.

GDF15 has been reported to be protective toward chronic complications that arise from old age or diabetes (9, 23). Notably, mice with GDF15 deletion are susceptible to diet-induced obesity compared to controls, indicating the possible protective role of GDF15 against the severity of diet-induced obesity (24). Similarly, overexpression of GDF15 reduced body weight and augmented the metabolic profile in different animal models of disease (25). Thus, GDF15 clearly has a role in metabolic adaptation and acts potentially as a regulator of phenotypes of obesity that require further investigation. Our data is consistent with pre-clinical studies in mouse models, wherein circulating levels of GDF15 were observed as elevated in obese mice compared to lean controls (25). Furthermore, our findings are in agreement with reports indicating the association of elevated circulating levels of GDF15 with stress conditions such as myocardial injury or acute kidney injury (20, 26). We speculate the possibility that GDF15 in obese patients could be adaptive and reflect the nutrient overload-associated stress, metabolic, or tissue remodeling of both adipose and skeletal muscle. However, this potential protective mechanism of GDF15 could be short-lived and remains to be characterized or demonstrated experimentally, which is beyond the scope of the present study.

The association of elevated plasma GDF15 with cardiovascular diseases is well-established (12, 27). Moreover, the heart appears to be an important source of this cytokine (28), from multiple cell sources including the myocyte, which we reaffirm. However, which cells in the heart secrete this protein and what induces each cell type is unclear. Our findings are supported by work in rodent models that suggest cardiomyocytes secrete GDF15 following ischemia reperfusion (9, 29). Furthermore, the immunohistochemical expression of GDF15 was localized to the cytoplasm of cardiomyocytes in atrial tissues, the pattern is consistent with those observed by others in human and rodent cardiac muscles (28, 30). However, liver (31) or adipocytes (8) are also reported to secrete GDF15 under various cellular stress and/or experimental conditions, and we could not verify this in the present study. We acknowledge our study's inability of deciphering the exact contribution of GDF15 produced by non-cardiac tissues in our patients due to sampling constraints.

The majority of patients undergoing heart surgery had normal heart function (EF >50%, *n* = 75%) but some patients (46%) displayed signs and symptoms of HF also known as heart failure with preserved EF (HFpEF) before surgery. HFpEF is an important clinical condition that is poorly understood and associated with significant patient morbidity (32). HFpEF is often associated with inflammation (TNFα), cardiac fibrosis, and cardiac stress (natriuretic peptide expression). We were able to demonstrate a significant correlation between GDF15 expression and NT-proBNP, supporting a mechanistic link between cardiac remodeling, obesity and thus potentially HFpEF. It is suggested that GDF15 has anti-inflammatory effect (33) and its expression has been strongly linked to inflammation under several pathological conditions (9). Inflammation is also an important hallmark in the development and progression of HF with cardiac remodeling (34). GDF15 has been associated with cardiac fibrosis and its expression dramatically increases during cardiac stress (35). Thus, inflammation within cardiac tissues in response to HF represent important mechanisms that can act as the trigger for the synthesis and secretion of extracellular GDF15 into the plasma, and we cannot rule out that inflammatory cells may also be a potential source of GDF15. Notably in our study other markers of inflammation like NLR, Galectin 3, ST2, or TNFR2 were not as salient. We acknowledge that we did not test all possible inflammatory-related biomarkers but focused instead on more novel biomarkers that have recently been studied in cardiac patients (36). Our findings are limited by our small group of patients in which one could fully adjust for differences in clinical characteristics between groups, in particular, with regards to medications that could have effects on inflammation, structural or metabolic remodeling. Indeed, is has been reported that insulin sensitizing and angiotensin class inhibitors are able to reduce GDF15 (37). Detailed information on all pharmaceuticals was not available for analysis. Regardless, pre-operative standard of care was not sufficient to eliminate the association of GDF15 with poor outcomes. As such, the medical management strategy could still be informed by GDF15 and future studies seeking more aggressive treatment plans in patients with the 75th percentile of expression could be developed.

Despite these limitations, our findings suggest that the severity of obesity appears to be linked to elevated GDF15 and possibly relevant to structural, metabolic or inflammatory remodeling and adaptions pertinent to conditions such as HFpEF, given our clinical population and coordinate NTpro-BNP findings. GDF15 levels were seen over a wide range of values. We chose to group patients based on the 75th percentile to define elevated levels of GDF15 compared to lower levels, and this could assist other small centers or contribute to meta-analysis in future studies or study designs. Our approach was largely based on how biomarkers are often used clinically (38, 39). That said, the 75th percentile was arbitrarily chosen and the values should be approached with caution for this reason but it allowed us to appreciate at what level one could expect to see significant association with a hard clinical outcome, mortality. We cannot say with certainty that the therapeutic objective is a reduction of GDF15, without more mechanistic insight into whether it is adaptive or maladaptive with respect to disease trajectory or post-operative recovery. A prospective trial and broader population sampling is thus required. Recently, this was highlighted as a need in a meta-analysis, corroborating our findings for risk of mortality in heart failure and acute coronary syndrome (40). More importantly, although obese and non- obese patients had similar outcomes, GDF15 was able to identify patients at risk of worse outcomes over time with higher GDF15 showing a worse survival 24 months post-surgery independent of obesity. Our findings are supported by a body of literature suggesting an association between GDF15 and mortality using different levels/quartiles of GDF15 (12). Importantly, our findings do not mean that GDF15 is itself detrimental or diagnostic but does suggests its greater value as a salient prognostic marker, even in small unadjusted populations. This could help inform decision making in the art of medicine for individual case management. Further work is needed to determine if GDF15 expression is meant as a protective measure to limit the impact of stress on the organism as suggested by others (41), perhaps by limiting the inflammatory milieu observed during obesity. In summary, our finding highlights the potential role of GDF15 as a marker of stress in obese individuals who are at greater risk of HFpEF and mortality long term. We propose that GDF15 may represent an important biomarker that would allow us to better determine which obese patients are at greatest risk of adverse events but this remains to be proven definitively (8, 42).

## Data Availability Statement

All datasets generated for this study are included in the article/Supplementary Material.

## Ethics Statement

The studies involving human participants were reviewed and approved by Horizon Health Network Research Ethics Board (REB). The patients/participants provided their written informed consent to participate in this study.

## Author Contributions

SS: all tissue analysis, generation of figures and writing, formatting, and revisions of the manuscript. SLe and IH: ELISA and interpretation of results. JM: critical in the study design and responsible for acquiring funding. JBM: all statistical analyses. CA: obtaining study approvals and samples. SLu, AH, KB, PK, and TP: critical inputs for the project and revision of the manuscript. J-FL: acquired the funding in collaboration with JM, wrote the manuscript with SS, and was the principal investigator of the work. All authors: read and approved the final manuscript.

## Conflict of Interest

The authors declare that the research was conducted in the absence of any commercial or financial relationships that could be construed as a potential conflict of interest.

## Supplementary Material

Supplementary Figure 1 **(A)** Atrial GDF15 showed low mRNA expression. mRNA expression was normalized to YWHAZ. **(B)** Atrial GDF15 mRNA did not show a significant association with plasma GDF15 **(i)** or atrial GDF15 protein expression **(ii)**.

Supplementary Table 1 Patient characteristics when divided on the basis of high and low GDF15.

## Abbreviations

ACT: Activated Clotting Time
BMI: Body Mass Index
CABG: Coronary Artery Bypass Graft
CCL2: C-C Motif Chemokine Ligand 2
CHF: Chronic Heart Failure
Ct: Cycle threshold
DAB: 3,3'-diaminobenzidine tetrahydrochloride
EF: Ejection Fraction
GDF15: Growth Differentiation Factor 15
HF: Heart Failure
HFpEF: Heart Failure with preserved Ejection Fraction
HRP: Horse Radish Peroxidase
IL-18: Interleukin 18
IQR: Inter Quartile Range
LVEDP: Left Ventricular End-Diastolic Pressure
MMP: Matrix Metalloproteinase
NBHC: New Brunswick Heart Centre
NLR: Neutrophil-to-Lymphocyte Ratio
NTproBNP: N-Terminal pro b-type natriuretic peptide
NYHA: New York Heart Association
PVD: Peripheral Vascular Disease
qPCR: Quantitative Polymerase Chain Reaction
RNA: Ribonucleic Acid
SICU: Surgery Intensive Care Unit
ST2: Suppression of Tumorigenicity 2
TC: Total Cholesterol
TG: Triglyceride
TGF-β: Transforming Growth Factor beta
TNFR2: Tumor Necrosis Factor Receptor 2
VEGFA: Vascular Endothelial Growth Factor A
Take-home message: GDF15 is significantly associated with obesity severity but unlike obesity, appears better at identifying patients with worst survival at 2 years after cardiac surgery.
