# Supplementary figures and images for: Serum GDF15, a Promising Biomarker in Obese Patients Undergoing Heart Surgery
Source: Front Cardiovasc Med. 2020 Jun 24;7:103. doi: 10.3389/fcvm.2020.00103 (PMC7327098; doi:10.3389/fcvm.2020.00103)

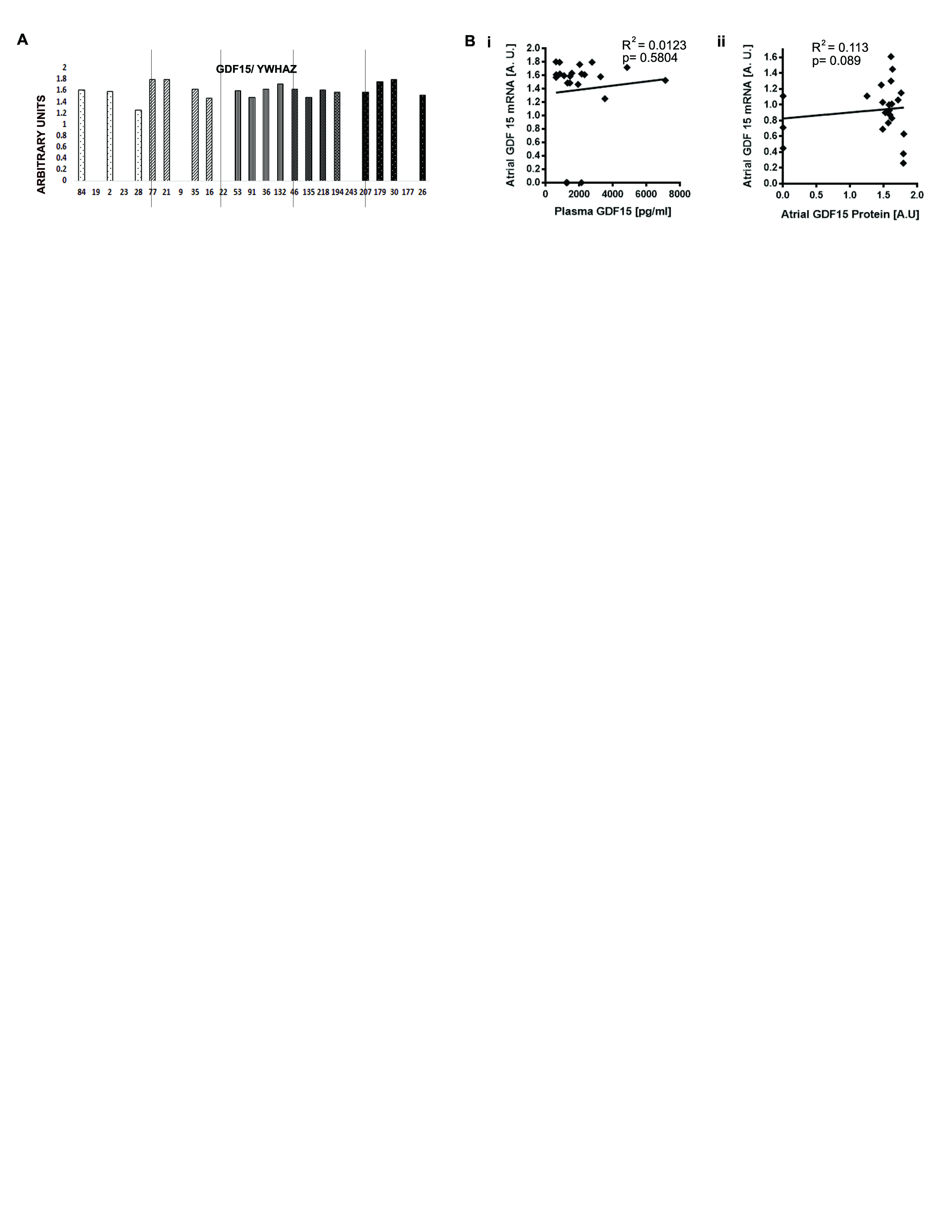

Supplement: Supplementary Figure 1 — (A) Atrial GDF15 showed low mRNA expression. mRNA expression was normalized to YWHAZ. (B) Atrial GDF15 mRNA did not show a significant association with plasma GDF15 (i) or atrial GDF15 protein expression (ii). [file Image_1.tif]
