# Supplementary material for: Serum GDF15, a Promising Biomarker in Obese Patients Undergoing Heart Surgery
Source: Front Cardiovasc Med. 2020 Jun 24;7:103. doi: 10.3389/fcvm.2020.00103 (PMC7327098; doi:10.3389/fcvm.2020.00103)
Supplement: Supplementary Table 1 — Patient characteristics when divided on the basis of high and low GDF15. [file Table_1.docx]

**Supplementary table 1: Patient characteristics when divided on the basis of high and low GDF15**

| **Characteristic** | **GDF15** | | | | **p-value** |
| --- | --- | --- | --- | --- | --- |
|  | **High** | | **Low** | |  |
|  | **n=24** | | **n=56** | |  |
| Age, years | 66 | ± 6 | 63 | ± 8 | 0.06 |
| Female sex | 4 | (17) | 12 | (21) | 0.77 |
| BMI class |  |  |  |  |  |
| Normal | 4 | (17) | 11 | (20) | **< 0.001** |
| Pre-obese | 2 | (8) | 21 | (38) |  |
| Obese class 1 | 4 | (17) | 13 | (23) |  |
| Obese class 2 | 6 | (25) | 10 | (18) |  |
| Obese class 3 | 8 | (33) | 1 | (2) |  |
| Diabetes | 18 | (75) | 11 | (20) | **< 0.001** |
| Dyslipidemia | 18 | (75) | 38 | (68) | 0.71 |
| Hypertension | 23 | (96) | 38 | (68) | **0.02** |
| PVD | 3 | (13) | 3 | (5) | 0.36 |
| CVD | 3 | (13) | 5 | (9) | 0.69 |
| Renal failure | 1 | (4) | 0 | (0) | 0.30 |
| LVEF | 58 | ± 11 | 61 | ± 11 | 0.35 |
| Atrial fibrillation | 5 | (21) | 5 | (9) | 0.16 |
| NYHA class | 3 | (2–3) | 2 | (2–3) | 0.10 |
| 6MWT distance, m | 260 | ± 109 | 343 | ± 99 | **< 0.001** |
| Procedure |  |  |  |  |  |
| Isolated CABG | 12 | (50) | 32 | (57) | 0.84 |
| Isolated valve | 5 | (21) | 9 | (16) |  |
| Other/combined | 7 | (29) | 15 | (27) |  |
